# Costs Associated with Low Birth Weight in a Rural Area of Southern Mozambique

**DOI:** 10.1371/journal.pone.0028744

**Published:** 2011-12-12

**Authors:** Elisa Sicuri, Azucena Bardají, Betuel Sigauque, Maria Maixenchs, Ariel Nhacolo, Delino Nhalungo, Eusebio Macete, Pedro L. Alonso, Clara Menéndez

**Affiliations:** 1 Barcelona Centre for International Health Research (CRESIB), Hospital Clínic, Universitat de Barcelona, Barcelona, Spain; 2 CIBER Epidemiología y Salúd Publica (CIBERESP), Barcelona, Spain; 3 Manhiça Health Research Centre (CISM), Manhiça, Mozambique; 4 National Institute of Health and National Directorate of Health, Ministry of Health, Maputo, Mozambique; University of Florida, United States of America

## Abstract

**Background:**

Low Birth Weight (LBW) is prevalent in low-income countries. Even though the economic evaluation of interventions to reduce this burden is essential to guide health policies, data on costs associated with LBW are scarce. This study aims to estimate the costs to the health system and to the household and the Disability Adjusted Life Years (DALYs) arising from infant deaths associated with LBW in Southern Mozambique.

**Methods and Findings:**

Costs incurred by the households were collected through exit surveys. Health system costs were gathered from data obtained onsite and from published information. DALYs due to death of LBW babies were based on local estimates of prevalence of LBW (12%), very low birth weight (VLBW) (1%) and of case fatality rates compared to non-LBW weight babies [for LBW (12%) and VLBW (80%)]. Costs associated with LBW excess morbidity were calculated on the incremental number of hospital admissions in LBW babies compared to non-LBW weight babies. Direct and indirect household costs for routine health care were 24.12 US$ (CI 95% 21.51; 26.26). An increase in birth weight of 100 grams would lead to a 53% decrease in these costs. Direct and indirect household costs for hospital admissions were 8.50 US$ (CI 95% 6.33; 10.72). Of the 3,322 live births that occurred in one year in the study area, health system costs associated to LBW (routine health care and excess morbidity) and DALYs were 169,957.61 US$ (CI 95% 144,900.00; 195,500.00) and 2,746.06, respectively.

**Conclusions:**

This first cost evaluation of LBW in a low-income country shows that reducing the prevalence of LBW would translate into important cost savings to the health system and the household. These results are of relevance for similar settings and should serve to promote interventions aimed at improving maternal care.

## Introduction

More than twenty million low birth weight (LBW) babies are born every year in low-income countries [1]. LBW, defined by the World Health Organization (WHO) as weight at birth of less than 2.5 kg irrespective of gestational age, has adverse consequences on infant survival and physical and cognitive development [2], [3]. Very low birth weight (VLBW) babies, defined as weighing 1.5 kg or less at birth, have a high risk of death and disease during the first year of life [4]. In low-income countries about 60% of all infant deaths are reported in LBW babies [5]. Most of these deaths occur during the neonatal period when the risk of death is six times higher than in high-income countries [6]–[8]. LBW has been associated with an increased risk of respiratory and diarrhoeal disease [9]–[11], impaired growth and mental development, and poor outcomes in young adulthood [12]–[15]. HIV infection, malaria, malnutrition, and anaemia during pregnancy along with maternal younger age have been reported to be risk factors for LBW [16]–[19].

Increasingly, global efforts are being deployed to improve child survival, a key target of the Millennium Development Goals (MDGs) [20], [21], and LBW is a well known contributor to neonatal and infant mortality [8], [22]. In addition, the care of LBW babies represents an enormous burden for families and health systems, which may be particularly relevant in low-income countries because of the already stressed weak health systems. While there is wide evidence on costs associated with LBW in high-income countries [23]–[27], data on economic implications of care of LBW babies are scarce for middle-income countries [28], [29], and almost nonexistent for Sub-Saharan Africa. The only study carried out in this region reported that the costs of hospital care for VLBW infants were very high for the health system [30].

Estimates of the economic burden of public health problems that represent a huge toll on infant survival, and economic evaluations of effective interventions that improve this survival are crucial to guide health policies [31]. This study aimed at estimating the costs of health care of LBW babies for the health system and the household during the first year of life in a rural area of Southern Mozambique. In addition, the study assessed the burden of mortality associated with LBW as well as the relationship between weight at birth and costs of routine health care for the household.

## Methods

### Ethics Statement

The study was approved by the National Mozambican Ethics Review Committee and the Hospital Clinic of Barcelona Ethics Review Committee. Written informed consent was obtained from all participants involved in the study.

### Study area and population

The study was carried out in Manhiça, Maputo Province, a semi-rural area in Southern Mozambique. A Demographic Surveillance System (DSS) is run by the Centro de Investigaçâo em Saúde de Manhiça (CISM) and covers Manhiça town and surrounding villages, the Manhiça study area, with a total population under surveillance of over 80.000 inhabitants, which constitutes about 60% of the population of the Manhiça District. The main economic activities are subsistence farming and sugarcane and fruit processing. Most commercial activities are concentrated along the main road from Maputo to Beira. There are two main towns in the district, Manhiça and Xinavane, but most of the population lives in small dispersed villages. The climate is subtropical with a warm and rainy season between November and April, and a cool and dry season during the rest of the year. Geographical and demographic characteristics of the area have been described elsewhere [32].

Adjacent to the CISM is the Manhiça District Hospital (MDH), a 110 bed health facility, which provides preventive and curative services. A round-the-clock hospital-based clinical surveillance system has been operating in the area since 1997 and has been described in detail elsewhere [33]. In brief, all children attending the paediatric department are seen by project health personnel who administer a detailed standardized clinical and demographic questionnaire that documents signs and symptoms including birth weight. Eighty percent of pregnant women in this area have institutional delivery (Nhacolo A, personal communication). Before discharge from hospital delivery information on the newborn's anthropometric measurements and gestational age are registered onto standardized questionnaires. Infant and neonatal mortality rates for 2005–2006 in the area were 75 and 26 per 1000 live births, respectively (Nhacolo A., personal communication).

### Study design

Information on household costs for LBW babies was collected between July and November 2007 at the MDH through exit survey. Two sources of incremental cost were identified for LBW compared to non-LBW weight babies during the first year of life, i.e., costs for routine health care (incremental days of admission after hospital delivery and weighing visits) and costs for excess morbidity (hospital admissions and outpatient attendances). In order to measure costs incurred by the households, caretakers of LBW babies were invited to participate in the survey when leaving the hospital and if informed consent was signed a standardised questionnaire was administered. Additional information on direct cost (care and treatments received) and indirect costs for babies transferred to the Maputo Central Hospital (MCH) was obtained retrospectively through household visits.

Information on health provider costs was collected by interviewing clinical staff to illustrate routine health care and resources required to provide necessary health care for LBW babies at the MDH and MCH. Unit costs were obtained from previous estimates and data provided by the administration department of the MDH [34]. Prior to the calculation of costs associated with LBW, differences in morbidity between LBW and non-LBW weight infants were estimated from the clinical surveillance information collected from a cohort of children of a study carried out in the area [35]. DALYs due to excess mortality were calculated based on the evidence that LBW is a statistically significant risk factor for infant death in the area and it is, thus, responsible for incremental mortality compared to non-LBW weight [36].

#### Routine health care of LBW babies

The routine health care that LBW babies receive in Mozambique (as recommended by the WHO) depends on their weight at birth and lasts until the infant's weight reaches 2.5 kg [37]. All VLBW newborns (weight ≤1.5 kg) are transferred to the tertiary referral hospital in the capital (MCH) where they are admitted on average for up to 3 weeks if they have no further complications. LBW babies between 1.5 and 2 kg are admitted at the MDH for less than a week on average. Babies born with a weight between 2 kg and 2.5 kg are not admitted longer than non-LBW birth weight babies. About 30% of LBW babies with weight between 1.5 kg and 2 kg are transferred to the MCH due to clinical complications (Sigauque, personal communication). Many small babies referred to the MCH have respiratory distress syndrome which requires supplementary oxygen and in many cases surfactant therapies. Neonatal jaundice is treated with phototherapy. According to international guidelines, the follow up of LBW babies requires weekly visits to the health facility for weight control until they reach 2.5 kg. At these visits their health status is evaluated. All LBW babies receive iron supplementation and multivitamins for 3 months [37]. After hospital delivery, mothers of babies with weight at birth ≤2 kg are trained on kangaroo mother care (KMC) techniques [38].

#### Household Costs

The unit of analysis of costs associated with routine health care was the LBW baby. Costs associated with health care received until a baby reaches 2.5 kg of weight, were estimated [37]. The number of required visits at the health facility (N) was estimated according to previous estimates of daily weight gain in LBW babies [39], using the following formula:
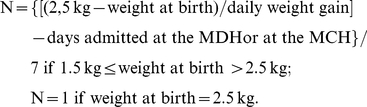
Costs associated with outpatient visits and hospital admissions during the first year of life were estimated as the sample average cost of a hospital admission or of an outpatient attendance. The inclusion of these costs in the estimate undertaken in this study was conditioned on significant positive difference in the number of visits or admissions among LBW compared to non-LBW babies during the first year of life [36].

Both for routine health care (incremental days of admission after hospital delivery and weighing visits) and excess morbidity (hospital admissions and outpatient attendances), direct and indirect costs for LBW were estimated. Direct costs included: transportation to and from the health facility, expenses incurred while staying at the hospital (food, telephone, etc), other travel cost incurred by caretakers while the baby was admitted to hospital, as well as accommodation costs for nights spent away from home. A fixed user fee of 0.15 US$ was paid by families to the MDH. Iron and multivitamin supplementation were given for free at the pharmacy of the MDH. No transportation costs to and from the hospital due to the delivery were included. Incremental costs of admission after hospital delivery were considered to be zero if the LBW baby was admitted at the MDH for just one night, as these costs were assumed to be associated with delivery rather than with LBW itself. The indirect costs calculated were income or welfare losses incurred by caretakers because of their children's need for care. Self-reported income losses, experienced by caretakers with a formal and remunerated occupation, were included in the analysis. On the other hand, welfare losses borne by caretakers without a formal and remunerated occupation (housewives) were conservatively estimated by multiplying the amount of time spent away from their main activity because of child's illness times the average national minimum statutory monthly wage in force in Mozambique at the time of the study (37 US$) [40].

#### Health system costs

Costs to the health system arising from routine care provided to LBW babies were calculated with reference to a target of 1000 live births at the MDH, assuming 12% and 1% of LBW and VLBW respectively in the area ([35], Nhacolo A, personal communication). The cost of a routine visit at the MDH was estimated as the monetary value of the proportion of health workers' time spent in this activity. The calculation of costs associated with hospital admissions and outpatient attendances was based on estimates of unit costs at Level I and II health facilities produced by the Ministry of Health [34]. All costs were confirmed with the administrative staff of the MDH and updated to 2007 levels using the average annual rate of inflation [41]. Health system costs for routine health care of LBW babies were calculated as the sum of costs of incremental hospital admission days after delivery and weighing visits. To calculate incremental hospital admission after delivery and weighing visit costs, unit costs were multiplied by the average number of nights spent at hospital and the average number of weighing visits, respectively. The cost of iron and multivitamin supplementation was added to this estimate [42]. It was estimated that, on average, it takes 7 minutes per baby for a nurse to provide training on KMC techniques. Total cost per newborn for such training was estimated as the value of 7 minutes of a nurse's time. Transportation costs of VLBW newborns to the MCH were estimated taking into account the relevant proportion of salary of a driver (approximately 3 hours), plus the cost of fuel necessary to cover the distance (80 km) from Manhiça to Maputo [43].

Costs per bed/day at the MCH were estimated to be 3 times higher than at the MDH because of higher availability and use of resources in this tertiary health facility. Tables 1 and 2 show the input variables for the cost to the household and the health system, respectively.

**Table 1:** Household costs inputs.

Inputs	Distribution	Low estimate	Best estimate	High estimate	Sources
Daily weight gain per weight range (grams)	Triangular	Blond et al. [39] minus 25%	Taken from Blond et al [39]	Taken from Anchieta et al [44]	[39], [44]
Individual direct and indirect costs (routine health care) (US$)	Triangular	Best estimate minus 25%	Estimate based on individual observations	Best estimate plus 25%	Our survey
Average incremental cost for admission after delivery (US$)	Triangular	17.44	41.15	64.85	Our survey
Average cost for routine weighing visits (US$)	Triangular	3.10	12.51	21.91	Our survey
Individual direct and indirect admission costs (US$)	Triangular	Best estimate minus 25%	Estimate based on individual observations	Best estimate plus 25%	Our survey

**Table 2:** Health system costs inputs.

Inputs	Description	Distribution	Low estimate	Best estimate	High estimate	Source
Iron supplementation	Quantity	Point estimate	NA	60 mg of iron,¼ tablet for 90 days	NA	Sigauque, pc^a^
	Unit cost (US$)	Triangular	0.0013	0.0072	0.0171	[42]
Multi vitamins	Quantity	Point estimate	NA	1 table for 90 days	NA	Sigauque, pc^a^
	Unit cost (US$)	Triangular	0.0018	0.0044	0.0777	[57]
Transportation to referral hospital	Price of 1 litre fuel (US$)	Triangular	0.60	1	1.35	[43]
	Distance (Km) Manhiça - Maputo	Point estimate	NA	80	NA	NA
	Time taken to cover distance (hours)	Point estimate	NA	3	NA	NA
	Hourly salary of a driver (US$)	Point estimate	NA	0.27	NA	[39]
Admission after delivery at district hospital	Cost of one bed/day (US$)	Point estimate	NA	17.28	NA	[34]
	Number of nights admitted	Uniform	2	NA	4	Interviews
Admission at referral hospital	Cost of one bed/day (US$)	Point estimate	NA	51.84	NA	[34] and interviews
	Number of nights admitted	Uniform	14		21	Interviews
Routine weighing visits	Monthly wage of 1 nurse (US$)	Point estimate	NA	324	NA	[34] and interviews
	Time spent for the visit (minutes)	Point estimate	NA	5	NA	Interviews
	Times infants taken to visit (N)	Individually determined	NA	NA	NA	Our estimate
Hospital admission all causes	Cost of one bed/day (US$)	Point estimate	NA	17.28	NA	[34] & interviews
	Number of nights admitted	Uniform	NA	6	NA	Our estimate
KMC^b^	Average time to teach KMC^b^ (minutes)	Triangular	5	7	15	Interviews
	Cost of nurse per minute (US$)	Point estimate	NA	0.03375	NA	[34]
	Proportion of newborns with the need of KMC^b^	Triangular	NA	0.5	NA	Interviews
LBW prevalence	Proportion of births that are LBW^c^ (less than 2.5 kg)	Triangular	0.10	0.12	0.14	[35]
VLBW^d^ prevalence	Proportion of births that are VLBW^d^ (equal or less than 1.5 kg)	Triangular	0.008	0.01	0.012	Nhacolo, pc^a^

a Personal Communication;

b Kangaroo Mother Care;

c Low Birth Weight;

d Very low birth weight.

#### Data analysis

Probabilistic sensitivity analysis (Monte Carlo simulation) was carried out to allow for uncertainty around cost estimates (Tables 1 and 2). A range of ±25% was used to represent variability around mean values. Daily weight gain was considered as a probability distribution [44]. The Metical/US$ exchange rate was also treated as a probability distribution [45].

The relationship between total household cost and birthweight was estimated using a log-linear regression model. The dependent variable was the logarithm of total household cost associated with routine health care until an infant reaches 2.5 kg. Logarithmic transformation of costs was carried out because of distribution skewness [46]. The independent variables were a constant term and weight at birth, measured in grams.

Poisson regression model was used to quantify the incremental number of visits or admissions experienced by LBW compared to non-LBW weight babies only after assessing their statistical significance.

DALYs due to premature death were calculated both for a target of 1000 live births and for the number of live births occurred in the area during the year 2007. It was estimated that out of 1000 babies born in Manhiça, 120 are LBW, and of these, 10 are VLBW. The case fatality rates for LBW and for VLBW compared to non-LBW weight babies during the first year of life were estimated to be 12% and 80%, respectively (Sigauque B., personal communication) [36]. Years of Life with Disability (YLDs), for the first weeks of life, were included in DALYs calculation, applying a duration disability of 2 to 21 days (uniform distribution) and a disability weight of 0.256 for treated low birth weight sequelae. DALYs were calculated with reference to a global life expectancy (both sexes) for the year 2007 of 68 years [47], age-weighted using the standard age weighting function [48], and discounted at 3%.

## Results

### Baseline characteristics of study subjects

Fifty five caretakers of LBW babies were interviewed after hospital delivery, and another 32 at attendance to the routine weighing visit (Table 3). Mean birth weight was 2.26 kg, and 69% were females. Only 1 newborn was VLBW (≤1.5 kg). The average number of days admitted to the MDH and the average number of routine weighing visits were 3.44 and 2.13, respectively. The only newborn transferred to the MCH was admitted for 17 days before dying. Forty six caretakers of babies born with LBW were identified and interviewed at the time of hospital discharge or at outpatient attendance for an acute illness episode. The two main causes of hospital admission were respiratory infections and malaria, representing 33.3% and 27.0% of all admissions in the sample. Most caretakers were the infant's mother and the majority of them were housewives.

**Table 3:** Baseline characteristics of the study subjects.

			Frequency (N)	Percentage (%)
Evaluation of routine care costs of LBW babies	Newborns' gender	Male	27	31.0
		Female	60	69.0
	Place of interview	Routine admission	55	63.2
		Routine visit	32	36.8
	Caretaker's occupation	Housewife	73	83.9
		Seller	2	2.3
		Other	11	12.6
		Missing	1	1.2
	Caretaker's relation with newborn	Mother	87	100.0

			Mean	SD^a^
	Birthweight		2.26	0.40
	Number days admitted		3.44	2.99
	Number of routine visits		2.13	1.23

			Frequency (N)	Percentage (%)
Evaluation of LBW admission costs during the 1^st^ year of life	Newborns' gender	Male	21	46.0
		Female	25	54.0
	Caretaker's occupation	Housewife	39	84.8
		Seller	2	4.3
		Other	1	2.17
		Missing	4	8.7
	Caretaker's relation with newborn	Mother	43	93.48
		Other	3	6.52
	Main diagnosis	Respiratory disease	16	33.3
		Malaria	13	27.0
		Meningitis	2	4.1
		Dermatitis	2	4.1
		Gastroenteritis	1	2.2
		Anaemia	1	2.2
		Other	11	27.1

			Mean	SD^a^
	Number of days admitted		5.80	5.26

a Standard Deviation.

### Costs and DALYs

**Figure 1:**
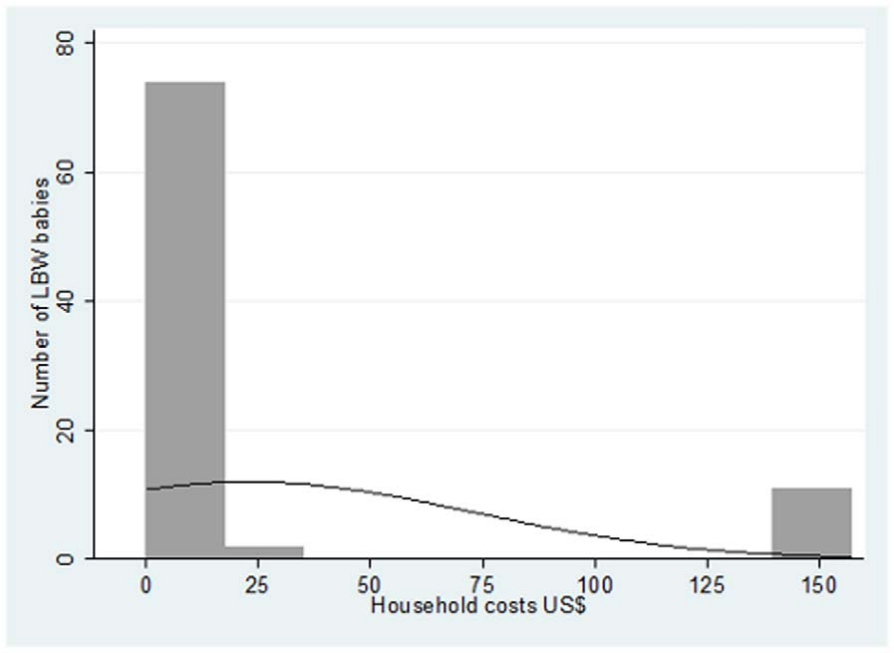
Household costs distribution for routine health care of low birth weight babies. These costs include incremental days of admission after hospital delivery and weighing visits.

The mean value of household cost, both direct and indirect, associated with routine health care for a LBW baby was 23.04 US$. The distribution of household cost was positively skewed with a median value of 4.06 US$, and minimum and maximum values of 0.19 and 156.86 US$, respectively (Figure 1). The maximum value of 156.86 US$ is due to referral to the tertiary hospital. Household costs assessed through Monte Carlo simulations were 24.12 US$ (CI 95% 21.51; 26.26).

The association between household costs for routine health care and weight at birth is represented by the following equation: Log (household costs) = 13.58–5.30*weight at birth. The results of the regression analysis suggest that an increase of 100 gr in the average weight at birth of a LBW baby would result in a reduction of 53% of the average cost to the household derived from routine health care.

**Figure 2:**
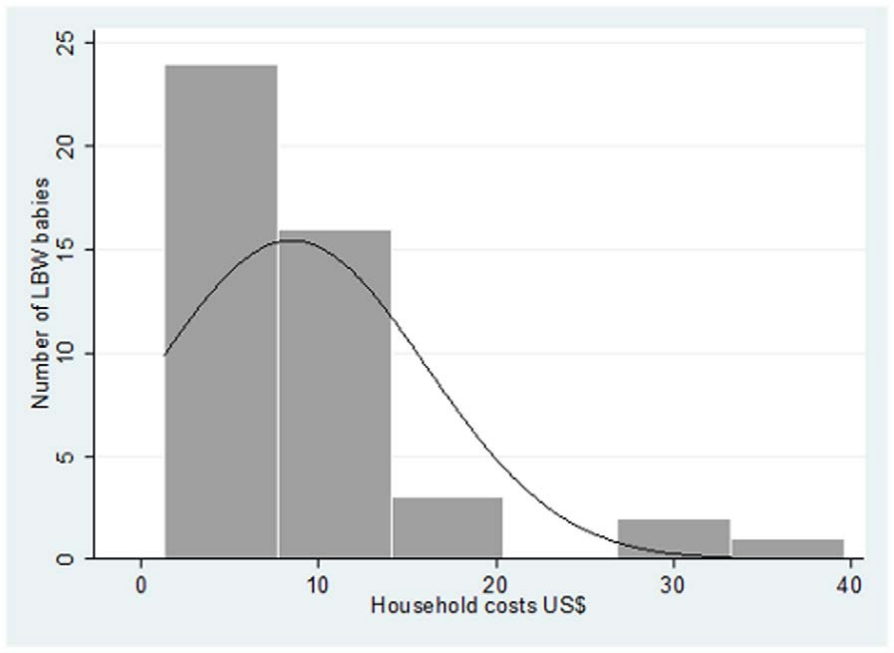
Distribution of the incremental costs to the household due to hospital admission of low birth weight (LBW) babies during the first year of life.

During the study 1004 babies were born alive at the MDH and were followed up for one year through the clinical and demographic surveillance systems in place in the area. Information on birthweight was registered for 990 of them (98.6%), and of these 117 were LBW babies (12%) [36]. There was no statistically significant difference in the number of outpatient attendances between LBW and non-LBW weight babies during the first year of life. Excluding deaths, 93.1% of non-LBW weight babies and 95.5% of LBW babies attended the MDH as outpatients at least once [Chi^2^(17) = 11.00; p = 0.856]. Including deaths, the percentage of outpatient attendance was 92.44% for non-LBW weigh babies and 84.6% for LBW babies [Chi^2^(17) = 19.78; p = 0.285]. However, the number of hospital admissions differed significantly between LBW and non-LBW weight babies. Excluding deaths, 21.3% of non-LBW weight babies and 34.4% of LBW babies were admitted at least once [Chi^2^(3) = 15.00; p = 0.002]. Including deaths, this percentage was 23.5% for non-LBW weigh babies and 37.6% for LBW babies [Chi^2^(3) = 11.93; p = 0.008]. LBW babies were expected to have a rate of hospital admissions during the first year of life 1.52 times greater than non-LBW babies (p = 0.007). Incremental costs associated with excess morbidity were thus calculated only on hospital admissions, based on one additional episode of admission for LBW compared to non-LBW weight babies. Incremental costs to the households due to hospital admission during the first year of life were 8.50 US$ (CI 95% 6.33, 10.72) of which 75% were indirect costs (Figure 2).

Based on Monte Carlo simulations, the total estimated health system costs for routine health care of LBW babies for a target of 1000 babies born in Manhiça, of which 120 were LBW and of these 10 were VLBW, were 50,953.68 US$ (CI 95% 41,460.00; 61,070.00) (Table 4). Incremental costs of admission due to LBW after hospital delivery at the referral tertiary hospital and at the MDH were 29,937.60 US$ (CI 95% 21,830.00; 38,760.00) and 4,145.43 US$ (CI 95% 2,130.00; 6,360.00), respectively. Incremental hospital admission costs due to excess morbidity during the first year of life were 11,612.16 US$ (CI 95% 10,002.00; 13,221.00). Transportation to the referral hospital cost 4,869.48 US$ (CI 95% 3,230.00; 6660.00), almost 10% of the total cost. Health system costs associated with LBW for the live births occurred during the year 2007 in the study area (N = 3,322) were 169,957.61 (CI 95% 144,900.00; 195,500.00).

**Table 4:** Health system costs and DALYs^b^ due to LBW^a^ in Manhiça (Mozambique).

Health system costs associated with LBW		
Costs for a target of 1000 live births^c^	US$ 2007	95% Confidence Interval
Iron supplementation	19.52	(5.84; 40.56)
Multivitamins	301.87	(43.00; 716.00)
Admission after delivery at district hospital	4,145.43	(2,140.00; 6,330.00)
Transportation to referral hospital	4,869.48	(3,230.00; 6,660.00)
Admission to referral hospital	29,937.60	(21,830.00; 38,760.00)
Routine health visits	49.71	(34.30; 70.50)
All causes admission during the first year of life	11,612.16	(10,002.00; 13,221.00)
Training of kangaroo mother care	17.13	(11.55; 23.97)
TOTAL	50,953.68	(41,460.00; 61,070.00)
TOTAL for the 3,322 live births occurred during the year 2007 in the study area	169,957.61	(144,900.00; 195,500.00)

DALYS^b^ due to death associated with LBW^a^	
For a target of 1000 live births	For the 3,322 live births occurred during the year 2007 in the study area
826.63	2,746.06

a Low Birth Weight.

b Disability Adjusted Life Years.

c Of 1000 live births at the Manhiça District Hospital, 120 are of LBW and 10 are of very LBW.

Applied to the target population of 1000 newborns considered in the analysis, it was estimated that 8 out of 10 VLBW and 17 out of 110 LBW would die during the first year of life. The overall DALYs due to death during infancy due to LBW were 826.63, of which 257.44 are attributable to VLBW babies. Considering 3,322 live births, the overall DALYs due to LBW in the study area were 2,746.06.

## Discussion

To our knowledge, this is the first economic evaluation of the costs associated with LBW in rural areas of low-income countries, and shows that LBW carries a significant economic burden both for the health system as well as for the families.

Health system costs during infancy associated with LBW babies among the 3,322 live births occurred in the study area during the year 2007 were almost 4 times higher than the average yearly national public health expenditure for the Expanded Programme on Immunization budgeted for the period 2007–2012, also calculated for a target of 3,322 live births [49]. Households also bore a significant economic burden, being the incremental LBW costs per child for LBW care more than 16 times higher than the cost, estimated in the same area, of an episode of malaria in a child [50]. Household costs for routine heath care would decrease by 53% for each 100 gr increase in birth weight, while avoiding deaths associated with LBW per 1000 live births, would save a significant amount of DALYs.

While incremental costs associated with LBW were presented for two cohorts of 1000 and 3,322 babies, it is important to mention the average costs both per LBW and non-LBW babies. For instance, non-LBW term newborns with an eutocic delivery are admitted less than 24 hours (cost of 1 bed/day from Table 2 = US$ 17.28) and, on average, 0.7 times during the first year of life for an average 6 nights (US$17.28*6). Therefore, the average costs associated with each non-LBW baby is US$ 121 (17.28+17.28*6). Being the estimated cost associated with LBW for a target of 1000 live births US$ 50,953.68 and considering that 120 babies out of 1000 are of LBW, the incremental cost per LBW baby is, on average, US$ 425 (50,953.68/120). Thus, the average cost per LBW baby is US$ 546 (425+121), 4.5 times higher than the cost per non-LBW baby.

We observed a gender imbalance, being 69% of LBW babies females and 31% males. This imbalance may be explained by the significant association between female gender and LBW observed among newborns in the area, and by the existence of a trend towards increased neonatal mortality among male LBW babies compared to female LBW babies [51].

The case fatality rate of VLBW babies in the area is 80% with these deaths occurring during the first month of life and thus, contributing to neonatal mortality. Through the comparison between DALYs due to the death of VLBW babies estimated in this study (257.44) and DALYs from all neonatal mortality in the area (836.68), it can be estimated that VLBW contributes to more than one third of the burden of neonatal mortality in the area. Thus, any intervention to reduce VLBW would have a significant impact in reducing neonatal mortality. Similarly, any intervention aimed at reducing the incidence of LBW would generate important cost savings to the families and the health system (apart from the benefit to the children themselves). LBW is mainly the consequence of poor maternal health; therefore improving the effectiveness of the already well attended ANC services by avoiding missing opportunities is an essential step to take to reduce the prevalence of LBW [52]. For instance, the use of point of care diagnostic tests at the ANC, followed by adequate treatment, against frequent causes of LBW, is likely to have an impact decreasing this burden.

Only one previous study in Africa made an assessment of the economic costs associated with LBW [30]. This was a tertiary level hospital-based study of costs associated with VLBW babies (N = 24) admitted to the intensive care unit in an urban area of Nigeria. Household costs were not calculated directly from the study sample. The results of the present study highlight the fact that costs associated with LBW are not restricted to those of sophisticated hospital care provided to VLBW babies.

Household cost distribution for routine health care appeared positively skewed, with one high value of 156.86 US$ (which included the cost of referral to the tertiary hospital), while the majority of the estimated costs were concentrated around similar lower values. Skewness of cost distribution is a well known issue and both probabilistic sensitivity analysis and logarithmic transformation were used to resolve it [46]. Interestingly, in the case of LBW routine health care, skewness of cost distribution strictly depended on weight at birth, with few higher costs corresponding to the few cases of VLBW, and with the majority of lower costs concentrated around the highest frequency of higher birth weight values. In the estimate of household costs, it is of note that the frequency of VLBW in the sample studied reflects the prevalence of VLBW in the area.

The estimated relationship between household costs for routine health care and weight at birth may appear as a tautology as birth weight was the main variable used to estimate household costs. However, the aim of the estimated regression between household costs and weight at birth was not to assess the existence of a relationship but rather to measure, through univariate analysis, the magnitude of such a relationship.

As it was done in a previous study, DALYs instead of costs, were used to measure the burden of death of VLBW newborns [53]. The reason behind this choice is related to the difficulties of estimating the economic value of lost lives [54]. However, it can be speculated that if such costs were considered, the economic burden of LBW would be much higher.

In this study the evaluation of the economic burden of LBW focus on short term costs and DALYs associated with LBW. In the health economics literature, the long term economic burden is usually measured considering costs associated with the lower educational and productive outcomes that are usually associated with LBW later in life. While some long term LBW costs estimates have been undertaken in high-income countries [55], these are extremely scarce in low-income countries [56]. Any measure of mortality or morbidity burden caused by LBW should include the long term disability associated with this health status at birth. Long term DALYs arising from preterm birth and not, thus, strictly relating to LBW, have been modelled in one study only [29].

Even just focusing on short term costs and DALYs, LBW represents an important economic burden that would justify the implementation of measures to reduce its prevalence. Further research, possibly cohort studies, should focus on the long term economic and health impact of LBW in low income areas in which lifelong disabilities are likely to impose a huge toll particularly on the households, as a consequence of fragile health systems lacking appropriate resources to manage chronic health impairments. Further research is also needed to explore intra-country variation of costs and DALYs associated with LBW. Knowledge of such a variation would assist scaling up LBW-related health interventions in the whole country. Although taking into account differences in health system organizations and LBW prevalence, extrapolation of these findings to other Sub-Saharan African countries might be possible for the similar resources available for the treatment and care of LBW babies.

## References

[1] UNICEFWHO (2004). Low Birthweight: Country, Regional and Global Estimates.

[2] McCormick MC (1985). The contribution of low birth weight to infant mortality and childhood morbidity.. N Engl J Med.

[3] Barker DJ (1995). The fetal and infant origins of disease.. Eur J Clin Invest.

[4] Ballot DE, Chirwa TF, Cooper PA (2010). Determinants of survival in very low birth weight neonates in a public sector hospital in Johannesburg.. BMC Pediatr.

[5] Guyer B, Hoyert DL, Martin JA, Ventura SJ, MacDorman MF (1999). Annual summary of vital statistics–1998.. Pediatrics.

[6] World Health Organization (2006). Neonatal and perinatal mortality: country, regional and global estimates.

[7] World Health Organization (2009). Global Health Statistics 2009.

[8] Lawn JE, Cousens S, Zupan J (2005). 4 million neonatal deaths: when? Where? Why?. Lancet.

[9] Victora CG, Barros FC, Kirkwood BR, Vaughan JP (1990). Pneumonia, diarrhea, and growth in the first 4 y of life: a longitudinal study of 5914 urban Brazilian children.. Am J Clin Nutr.

[10] Taylor B, Wadsworth J, Golding J, Butler N (1982). Breast-feeding, bronchitis, and admissions for lower-respiratory illness and gastroenteritis during the first five years.. Lancet.

[11] Lira PI, Ashworth A, Morris SS (1996). Low birth weight and morbidity from diarrhea and respiratory infection in northeast Brazil.. J Pediatr.

[12] Teles TP, Rodrigues T, Pereira A, Lopes C, Miguel C (1995). [Growth and development of children with low birth weight at their first birthday].. Acta Med Port.

[13] Ashworth A, Morris SS, Lira PI, Grantham-McGregor SM (1998). Zinc supplementation, mental development and behaviour in low birth weight term infants in northeast Brazil.. Eur J Clin Nutr.

[14] Bhutta AT, Cleves MA, Casey PH, Cradock MM, Anand KJ (2002). Cognitive and behavioral outcomes of school-aged children who were born preterm: a meta-analysis.. JAMA.

[15] Hack M, Flannery DJ, Schluchter M, Cartar L, Borawski E (2002). Outcomes in young adulthood for very-low-birth-weight infants.. N Engl J Med.

[16] Kramer MS (1987). Determinants of low birth weight: methodological assessment and meta-analysis.. Bull World Health Organ.

[17] Rollins NC, Coovadia HM, Bland RM, Coutsoudis A, Bennish ML (2007). Pregnancy outcomes in HIV-infected and uninfected women in rural and urban South Africa.. J Acquir Immune Defic Syndr.

[18] Brocklehurst P, French R (1998). The association between maternal HIV infection and perinatal outcome: a systematic review of the literature and meta-analysis.. British Journal of Obstetrics and Gynaecology.

[19] Guyatt HL, Snow RW (2004). Impact of malaria during pregnancy on low birth weight in sub-Saharan Africa.. Clin Microbiol Rev.

[20] United Nation http://www.un.org/millenniumgoals.

[21] Rajaratnam JK, Marcus JR, Flaxman AD, Wang H, Levin-Rector A (2010). Neonatal, postneonatal, childhood, and under-5 mortality for 187 countries, 1970–2010: a systematic analysis of progress towards Millennium Development Goal 4.. Lancet.

[22] Lawoyin TO (2001). Risk factors for infant mortality in a rural community in Nigeria.. J R Soc Promot Health.

[23] Russell RB, Green NS, Steiner CA, Meikle S, Howse JL (2007). Cost of hospitalization for preterm and low birth weight infants in the United States.. Pediatrics.

[24] Rogowski J (1998). Cost-effectiveness of care for very low birth weight infants.. Pediatrics.

[25] Boyle MH, Torrance GW, Sinclair JC, Horwood SP (1983). Economic evaluation of neonatal intensive care of very-low-birth-weight infants.. N Engl J Med.

[26] Lewit EM, Baker LS, Corman H, Shiono PH (1995). The direct cost of low birth weight.. Future Child.

[27] Tudehope DI, Lee W, Harris F, Addison C (1989). Cost-analysis of neonatal intensive and special care.. Aust Paediatr J.

[28] Cheah IG, Soosai AP, Wong SL, Lim TO (2005). Cost-effectiveness analysis of Malaysian neonatal intensive care units.. J Perinatol.

[29] Profit J, Lee D, Zupancic JA, Papile L, Gutierrez C (2010). Clinical benefits, costs, and cost-effectiveness of neonatal intensive care in Mexico.. PLoS Med.

[30] Tongo OO, Orimadegun AE, Ajayi SO, Akinyinka OO (2009). The economic burden of preterm/very low birth weight care in Nigeria.. J Trop Pediatr.

[31] Pedercini M, Barney G (2010). Dynamic analysis of interventions designed to achieve millennium development goals (MDG):. The case of Ghana Socio-Economic Planning Sciences.

[32] Alonso P, Saúte F, Aponte JJ, Gómez-Olivé FX, Nhacolo A (2002). Manhiça DSS, Mozambique. Population and Health in Developing Countries.

[33] Guinovart C, Bassat Q, Sigauque B, Aide P, Sacarlal J (2008). Malaria in rural Mozambique. Part I: children attending the outpatient clinic.. Malar J.

[34] Ministério da Saúde de Moçambique (MISAU)Austral Consultoria e Projectos Lda.Direcção de Planificação e Cooperação (2002).

[35] Menendez C, Bardaji A, Sigauque B, Romagosa C, Sanz S (2008). A randomized placebo-controlled trial of intermittent preventive treatment in pregnant women in the context of insecticide treated nets delivered through the antenatal clinic.. PLoS ONE.

[36] Bardaji A, Sigauque B, Sanz S, Maixenchs M, Ordi J (2011). Impact of malaria at the end of pregnancy on infant mortality and morbidity.. J Infect Dis.

[37] World Health Organization (2005). Hospital care for children: guidelines for the management of common illnesses with limited resources WHO, editor..

[38] Ruiz-Pelaez JG, Charpak N, Cuervo LG (2004). Kangaroo Mother Care, an example to follow from developing countries.. BMJ.

[39] Blond MH, Gold F, al Kadiry L, Rondeau C, Marchand S (1994). [Post-natal weight gain in the premature: the reference curves of Dancis (1948) can still be used].. Arch Pediatr.

[40] International Labour Organisation (2008). Global Wage Report 2008/09: Minimum wages and collective bargaining: Towards policy coherence..

[41] Kumaranayake L (2000). The real and the nominal? Making inflationary adjustments to cost and other economic data.. Health Policy Plan.

[42] Management Sciences for Health (2007).

[43] Petroleum Africa http://www.petroleumafrica.com/.

[44] Anchieta LM, Xavier CC, Colosimo EA, Souza MF (2003). Weight of preterm newborns during the first twelve weeks of life.. Braz J Med Biol Res.

[45] Oanda Currency Converter http://www.oanda.com.

[46] Briggs A, Gray A (1998). The distribution of health care costs and their statistical analysis for economic evaluation.. J Health Serv Res Policy.

[47] WHO Global Atlas of the Health Workforce (2009). Life expectancy at birth in years..

[48] Murray CJ, Lopez A (1996).

[49] Tyrrell A, Worrall E (2007).

[50] Conteh L, Sicuri E, Manzi F, Hutton G, Obonyo B (2010). The cost-effectiveness of intermittent preventive treatment for malaria in infants in Sub-Saharan Africa.. PLoS ONE.

[51] Menéndez C, Bardají A, Sigauque B, Romagosa C, Sanz S (2008). A randomized placebo-controlled trial of intermittent preventive treatment in pregnant women in the context of insecticide treated nets delivered through the antenatal clinic.. Plos One.

[52] Anya SE, Hydara A, Jaiteh LE (2008). Antenatal care in The Gambia: missed opportunity for information, education and communication.. BMC Pregnancy Childbirth.

[53] Sicuri E, Bardaji A, Nhampossa T, Maixenchs M, Nhacolo A (2010). Cost-effectiveness of intermittent preventive treatment of malaria in pregnancy in southern Mozambique.. PLoS ONE.

[54] Murphy KM, Topel RH (2006). The value of health and longevity.. Journal of Political Economy.

[55] Petrou S, Sach T, Davidson L (2001). The long-term costs of preterm birth and low birth weight: results of a systematic review.. Child Care Health Dev.

[56] Alderman H, Behrman JR (2004). Estimated Economic Benefits of Reducing Low Birth Weight in Low-Income Countries. Nutrition and Population (H N P) D I S C U S S I O N P A P E R.

[57] International Drug Price Indicator Guide (2007).

